# Control of Pollutants in the Trans-Boundary Area of Taihu Basin, Yangtze Delta

**DOI:** 10.3390/ijerph13121253

**Published:** 2016-12-17

**Authors:** Xiao Wang, Nikolaos Katopodes, Chunqi Shen, Hua Wang, Yong Pang, Qi Zhou

**Affiliations:** 1 College of Environment, Hohai University, Nanjing 210098, China; wangxiaohehai@163.com (X.W.); wanghua543543@163.com (H.W.); 2 Department of Civil and Environmental Engineering, University of Michigan, Ann Arbor, MI 48109, USA; ndk@umich.edu; 3 Department of Civil and Environmental Engineering, University of Wisconsin-Milwaukee, Milwaukee, WI 53211, USA; cshenjr@uwm.edu; 4 Key Laboratory of Integrated Regulation and Resources, Development of Shallow Lakes of Ministry of Education, Hohai University, Nanjing 210098, China; 5 College of Civil Engineering and Architecture, Tongling University, Tongling 244061, China; zq316249@sina.com

**Keywords:** Taihu Basin, trans-boundary area, total amount control of pollutant, trans-boundary effective control scope, water environmental capacity

## Abstract

This work focuses on pollution control in the trans-boundary area of Taihu Basin. Considering the unique characteristics of the river network in the study area, a new methodology of pollution control is proposed aiming at improving the water quality in the trans-boundary area and reducing conflicts between up and downstream regions. Based on monitoring data and statistical analysis, important trans-boundary cross sections identified by the regional government were selected as important areas for consideration in developing management objectives; using a 1-D mathematicmodel and an effective weight evaluation model, the trans-boundary effective control scope (TECS) of the study area was identified as the scope for pollutant control; the acceptable pollution load was then estimated using an established model targeting bi-directional flow. The results suggest that the water environmental capacity for chemical oxygen demand (COD), in order to guarantee reaching the target water quality standard in the TECS, is 160,806 t/year, and amounts to 16,098 t/year, 3493 t/year, and 39,768 t/year for ammonia nitrogen, total nitrogen, and total phosphorus, respectively. Our study method and results have been incorporated into the local government management project, and have been proven to be useful in designing a pollution control strategy and management policy.

## 1. Introduction

Taihu Basin, in the Yangtze Delta, is one of the most rapidly developing regions in China. The basin is composed of three provinces and one municipality, and contributes12% of China’s total GDP while occupying only 3.8% of the national land area [1,2]. Profiting from great accessibility due to its location, the trans-boundary area of the river network in Taihu Basin is a highly industrialized area. Over-urbanization and industrialization have caused deterioration of water quality in the area, and irreversible transfer of pollutants to other regions [3,4]. According to field investigations, several factors contribute to the inadequate management of the trans-boundary area of Taihu Basin:(1) since the 1980s, cities located in the trans-boundary area have benefited from free trade with various regions, causing a sharp increase in the speed of development in these areas, which has had major impacts on the local environment and caused numerous environmental problems. Among them, water pollution is regarded as extremely serious, with water quality in the river network threatened by a variety of pollution sources such as urban, industrial, residential, and agricultural [5,6]; (2) Against the current background of a lack of effective trans-boundary area management mechanisms, the non-cooperation of individual provinces in the basin has brought inherent environmental contradictions between cities along the rivers, and difficulties for jurisdictions trying to effectively regulate use of water resources in the trans-boundary area [7,8]; (3) The trans-boundary area of Taihu Basin, located in the estuary of Yangzi River, is close to East China Sea. Compared to other common trans-boundary areas, flow directions for the river network connected with East China Sea were strongly influenced by tidal movements. Pollution diffusion and the advection process in that complex hydrodynamic situation displayed much instability, which adds more difficulties for relevant research. All of these factors increase the difficulties associated with controlling the total amount of pollutant discharge in the trans-boundary area, and the responsibility of judgments regarding pollution problems in the area [9,10]. Overall, research on the trans-boundary area of the Taihu Basin river network has faced many technical and administrative difficulties.

There is worldwide concern regarding trans-boundary environmental problems, which have been increasing in prevalence and seriousness [11,12]. As a result, trans-boundary environmental management is becoming increasingly prioritized by environmentalists, litigators, and governments worldwide. According to trans-boundary environmental pollution research in the USA and Europe [13,14], lack of effective trans-boundary pollution control management is the main factor limiting water quality improvement in these areas. In addition, little effective management of the river network is currently available to deal with pollution in the trans-boundary area of the Taihu Basin. Thus, it is imperative to initiate research for management of the Taihu Basin trans-boundary area.

In this paper, the total amount control of pollutant (TACP), one of the most effective approaches to water environmental management [15,16,17], was selected as the management method. Targeting the aforementioned problems, a management methodology for the trans-boundary area in the plain river network of the Taihu Basin is proposed. Instead of using administrative cross sections as regional pollution control objectives (the approach widely used in previous studies),the trans-boundary cross sections were proposed to be the main factor of the TACP research, which is expected to play two roles: control objectives of regional pollution analysis, and provide indicators for dealing with contradictions between upstream and downstream political administrative regions. To secure the validity of pollution control based on minimal management consumption, TACP should be carried out in target districts that make clear contributions to boundary water quality. Given that the trans-boundary area in Taihu is an inaccurately defined area, a trans-boundary effective control scope (TECS), composed of several political and administrative districts in the trans-boundary area, was identified and clearly delineated. In view of the complex hydrodynamic situation and mass transport properties in the study area, a new method of calculating water environmental capacity was designed and the TACP was estimated, in order to determine the reasonable reduction rate for future management policies.

## 2. Research Materials

### 2.1. Study Area

**Figure 1:**
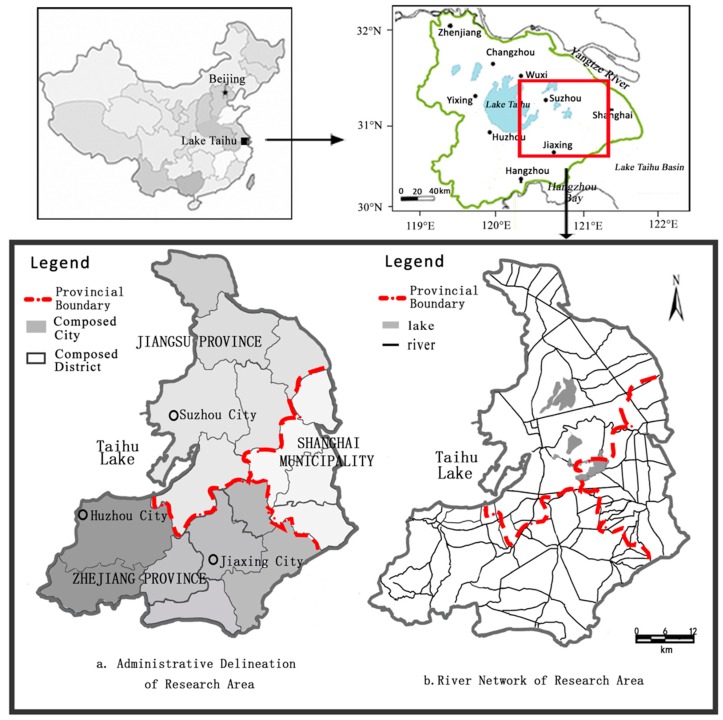
Map of Research Area.

As the core of the Yangtze Delta, Taihu Basin covers an area of 36,900 km^2^, and contains Jiangsu province, Zhejiang Province, and Shanghai Municipality. It has 400 km of provincial land boundaries composed of four parts: the boundary of Jiangsu and Zhejiang Provinces in the east (109 km) and the west (36 km), the boundary of Zhejiang Province and Shanghai Municipality (82 km), and the boundary of Jiangsu Province and Shanghai Municipality (114 km) [18,19]. The region located along these provincial boundaries in Taihu Basin covers an area of approximately 24,558 km^2^, accounting for 66.5% of the total area of the basin. The whole region can be divided into two parts: a hilly river area located to the west of Taihu Lake, and a plain river network area to the east. Since the water quality in the hilly river area, west of Taihu Lake, is always in an acceptable condition, in this paper, we targeted the east part of the trans-boundary area, the plain river network area. The targeted study area features a complex hydrological regime and pollution transport, leading to ineffective pollution control. The trans-boundary area contains three cities belonging to Jiangsu and Zhejiang Provinces, and five districts in Shanghai Municipality (Figure 1, Table 1), all of which influence and are affected by boundary water quality. These cities or districts are well-developed, populous, industrialized areas.

The study area has a high density of rivers (3.2 km/km^2^) [20], and according to field observations, many important trans-boundary rivers such as the Taipu, Former Grand, and Wusong Rivers, which transport a million tons of water across the boundary daily. These important trans-boundary rivers have multiple functions, such as water supply for drinking water, and for industrial and agricultural utilization. Given that these rivers are overloaded with pollutants, more attention should be paid to proposing an appropriate control plan [21].

### 2.2. Water Quality Monitoring and Evaluation at Boundary

**Figure 2:**
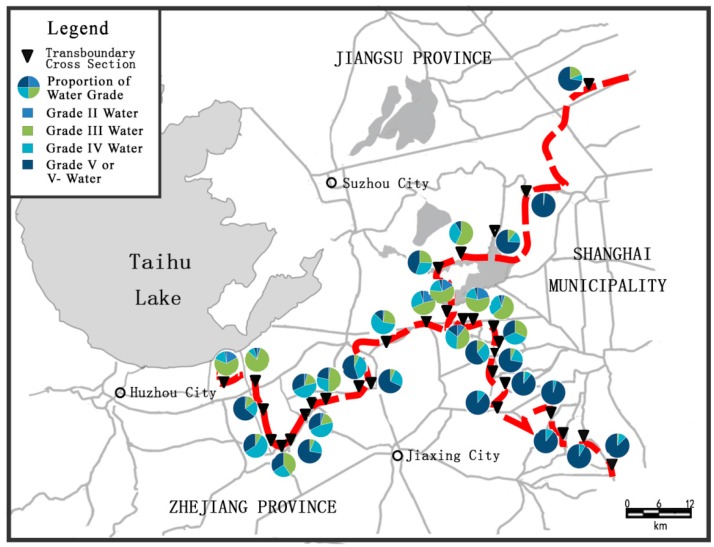
Spatial variation of water quality at trans-boundary cross sections in research area.

In the “Taihu Trans-Boundary Basin Bulletin” published by the Taihu Basin Authority of the Ministry of Water Resources, 31 important trans-boundary cross sections in the river network have been proposed as representative sections reflecting the boundary water environment. In this study, these cross sections were confirmed to be the central areas of consideration, and their corresponding water qualities were the main index of control management for the trans-boundary water environment. Eleven cross sections were located on the Jiangsu-Zhejiang provincial boundary, five on the Shanghai-Jiangsu provincial boundary, and the remainder on the Shanghai-Zhejiang provincial boundary (Figure 2).

**Figure 3:**
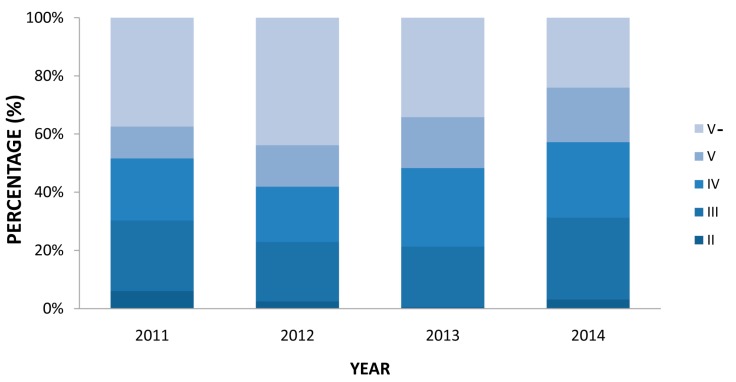
Temporal variation in water quality at boundaries from 2011 to 2014.

These 31 cross sections were monitored monthly by the Taihu Basin Authority of the Ministry of Water Resources since 2011, using three typical pollutant indices: chemical oxygen demand (COD_Cr_), ammonia nitrogen (NH_3_-N), and total phosphorus (TP). Based on the monitoring data of trans-boundary cross sections posted in the “Taihu Trans-Boundary Basin Bulletin” from 2011 to 2014, the variation of water quality in 31 sections can be analyzed in order to survey the boundary water quality status. According to the Environmental Quality Standard for Surface Water in China, the 31 sections can be graded from level II to level V−, with increasing deterioration of water quality. The spatial and temporal variation of water quality in the cross sections is shown in Figure 2 and Figure 3.

Figure 2 presents the proportion of each grade of water present at the cross sections. Almost 45% of important trans-boundary cross sections were always at level V or V−. On the other hand, less than 35% reached the requirements of level II or III, the minimum standards acceptable for groundwater. Water quality at the Shanghai-Zhejiang boundary has deteriorated the most, with 58% of readings being at level V or V–.

Figure 3 shows that the proportion of level V− water in the trans-boundary area increased from 37% in 2011 to 44% in 2012, and then declined by approximately 20%. Conversely, there was a rise in the proportion of water quality at levels II and III in 2013, before a decrease for three years until 2014, when the proportion of water quality at levels II and III accounted for 31% of the cross sections. These figures reflect that there has been a recent, though insufficient, improvement in trans-boundary water quality.

### 2.3. Pollution Sources in Study Area

Water pollution in the trans-boundary area is mainly caused by discharge of residential sewage, industrial sewage, livestock waste, and run-off from agricultural fields [22]. To quantify the pollution discharge in the study area, Emission Census Data (2011, 2013) collected by the Provincial Environmental Monitoring Center as well as the data from Statistical Yearbook (2011, 2013) [23,24] published by the Provincial Bureau of Statistics were used for the analysis of typical pollutants.

Discharge of typical pollutants for different administrative regions in 2011 and 2013 was estimated in order to discuss the annual and regional variation (Table 2). These data indicate that the amount of COD_Cr_ discharged in the total study area was 406,835 t in 2011, which decreased by 5.1% in 2013. Discharge of NH_3_ was 49,532 t in 2011, which then dropped by 5.5% in 2013. Similarly, the discharge amounts of TP and total nitrogen (TN) decreased by 11.9% and 8.2%, respectively, between 2011 and 2013. Although the amount of pollution discharged from Suzhou City in Jiangsu Province increased from 2011 to 2013, amounts from Jiaxing City and Huzhou City in Zhejiang Province and Shanghai Municipality declined between these two years. These water quality analyses results show that pollution control has had an impact on the improvement of boundary water quality.

**Figure 4:**
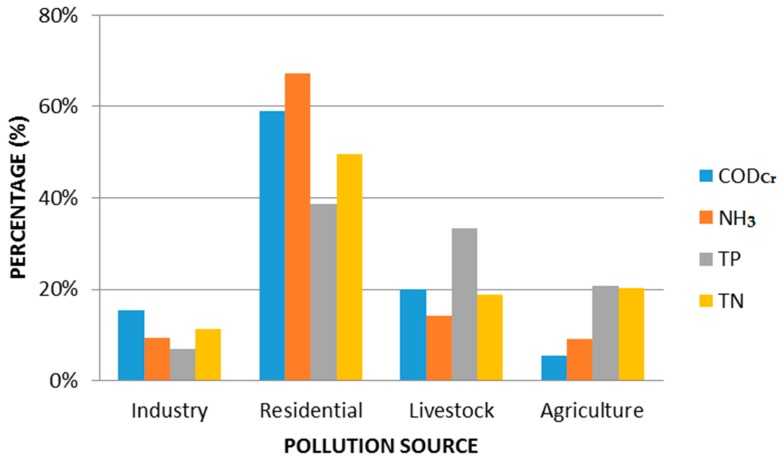
Contribution of different pollution sources in study area (2011–2013).

The typical pollutants discharged from different sources were estimated as well, and the contribution proportion of each pollution source is shown in Figure 4. Residential sewage was the dominant discharge source, contributing 58.8% of COD, 67.3% of NH_3_, 38.8% of TP, and 49.6% of TN. This is mainly attributed to the large population of the basin. In addition, some residential sewage, especially from rural areas, is currently not appropriately treated.

## 3. Research Method

### 3.1. Data Sources and Calculation Conditions

Data used in this study included both statistical information, such as the amount of multifarious pollution discharged, and monitoring data such as water quality and the hydrodynamics of the main rivers. All data were collected separately by various agencies belonging to different administrative management units: (1) the statistical pollution sources include 2151 industry pollution sources, 193 sewage treatment plants, and 1070 centralized animal farms, and other non-point sources such as agricultural fields, which all come from the census data carried out by provincial Environmental Monitoring Centers and the regional Bureau of Statistics; (2) the hydrodynamic data, such as flow rate and water level, of all the main rivers are from 65 hydrological stations that carry out daily monitoring in the study area, and belong to the Taihu Basin Authority of the Ministry of Water Resources; (3) the water quality data are from bimonthly monitoring of 78 water quality monitoring stations belonging to the provincial Environmental Monitoring Centers. Four pollution indices, COD, ammonia nitrogen (NH_3_-N), TP, and TN, were selected.

### 3.2. Delineation of the Trans-Boundary Effective Control Scope

The trans-boundary area of the plain river network in Taihu is a vaguely defined area. Proposing total pollution control management over a large, ill-defined area would increase the difficulty of carrying out the management. Thus, an appropriate scope for pollution control must be identified, and reduction plans scaled to this scope. In addition, to guarantee the accessibility of pollution control and the convenience of administrative management, the administrative division and distribution of administrative control units should be taken into consideration in the TECS delineation.

The TECS should consist of the area where pollution control will have a valid influence on the improvement of boundary water quality. This can be verified using the one-dimensional water quality equation and effect weight model. A one-dimensional steady-state water quality model was used to simulate the relationship between pollution sources and water quality, *c*, as follows (Equation (1)):

(1) $$c=\exp\left(-K\times \frac{x}{86400\times u}\right)\frac{C_{t}\times Q_{t}+Q_{0}\times C_{0}}{\left(Q_{0}+Q_{p}\right)}$$

where *c* is the pollutant concentration at the cross section at the provincial boundary; *K* is the degradation coefficient for the target pollutant; *u* is the velocity in the main stream; *x* is the calculated distance along the channel; *C*_0_ is the concentration at the incoming main stream; *Q*_0_ is the discharge of the main stream flow; *C_t_* is the concentration at the joining tributary flow; and *Q_t_* is the tributary flow discharge.

The effect weight model evaluates the degree of influence of pollution sources on water quality at the provincial boundary. The detailed equation can be expressed as follows (Equation (2)):

(2) $$\alpha =\frac{c_{i}}{c_{0}+c_{i}}$$

where *α* is the positive effect weight of pollution sources (%); *c_i_* is the pollutant concentration at the boundary calculated under the condition that pollutants along the channel were taken into consideration; and *c*_0_ is the solute concentration at the boundary calculated under the condition that all pollutants were ignored.

### 3.3. Calculating Water Environmental Capacity

Taking the characteristic of reversed flow observed in the study area into consideration, a new water environmental capacity calculation method targeted for trans-boundary area was proposed.

Firstly, as important trans-boundary rivers in TECS (Trans-boundary Effective Control Scope) have been traditionally divided into two segments by provincial boundary, trans-boundary cross sections located in the boundary of river segments were proposed to be the main control factors. Based on the well-known 1-D calculation equation [25], the environmental capacity of the trans-boundary river segment *i* in TECS characterized by bi-directional flow was calculated using the following equations (Equations (3)–(5)):

(3) $$W_{i}=\alpha \times W_{1i}+\beta \times W_{2i}$$

(4) $$C_{Ti}=\frac{W_{1i}+Q_{1i}\times C_{Ci}}{Q_{1}}\exp\left(-K\times \frac{x}{86400\times u_{1i}}\right)$$

(5) $$C_{Ci}=\frac{W_{2i}+Q_{2i}\times C_{Ti}}{Q_{2i}}\exp\left(-K\times \frac{x}{86400\times u_{2i}}\right)$$

where *W*_1i_ and *W*_2*i*_ are the calculated pollution loads of positive and negative flow, respectively, in river segment *i*; α is the rate of positive flow, and β is the rate of negative flow; *C_Ti_* is the concentration of river segment *i* at the trans-boundary cross section in calculation boundary; *C_Ci_* is the concentration of river segment *i* at the administrative cross section located in the other calculation boundary; *Q*_1*i*_ and *Q*_2*i*_ are the average discharges of positive and negative flow, respectively, in river segment *i*; and *u*_1*i*_ and *u*_2*i*_ are the average velocity of positive and negative flow, respectively, in river segment *i*.

If *C_Ti_* and *C_Ci_* are required to achieve the prescriptive target water quality according to the relevant plan, *W_i_* is the corresponding water environmental capacity of target river segment *i*, and thus it can be calculated.

Then, the water environmental capacity of the trans-boundary area was calculated using the following equation

(6) $$W=\frac{\sum_{i=1}^{n}W_{i}Q}{\sum_{i=1}^{n}Q_{i}}$$

where *W* is the corresponding water environmental capacity of the target area; *n* is the number of river segments in the target area, *Q* is the whole regional exchange flow of the target area; and *Q_i_* is the total exchange flow of river segment *i*.

## 4. Results and Discussion

### 4.1. Delineation of TECS

Referring to other research on control unit delineation based on effect analysis of pollution in Taihu Basin [26,27], the empirical value of effect weight α should be in the range of 60%–70%. Based on the statistical results of discharging pollution sources, and the monitoring data on the hydrodynamics and water quality of the river network, the corresponding range of river length, *x*, could be calculated using the effect weight and the 1-D water environment mathematical model. Combining this with the distribution of administrative districts and political management units in Taihu Basin allowed for the identification of effectively influencing districts along the provincial boundary, and delineation of TECS.

**Figure 5:**
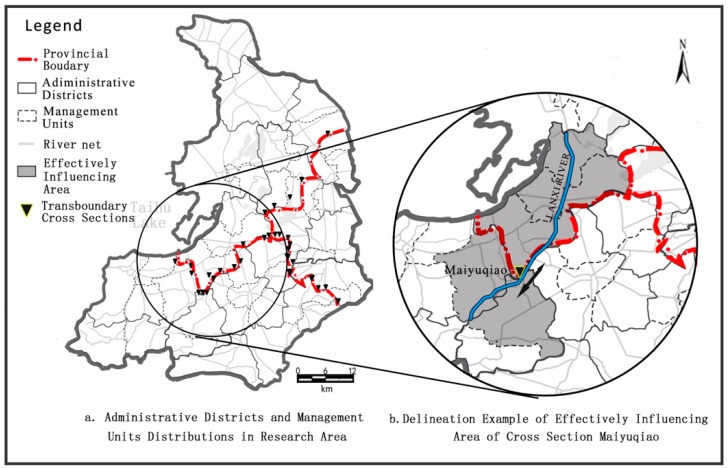
Example of trans-boundary effectively influencing area of trans-boundary cross section.

For example, a typical cross section, Maiyuqiao, located on the provincial boundary between Jiangsu and Zhejiang, is the management object of the trans-boundary river Jinghang Channel. Its main flow direction is from Jiangsu to Zhejiang Province. The average positive flow is 28.5 m^3^/s while the average negative flow is 11.2 m^3^/s. The maximum positive length that qualified for 60%–70% effect weight was calculated to be 26.5 km, and the maximum negative length was 7.2 km. Combining the distributions of administrative districts and management units around Maiyuqiao, the districts or units in dark, shown in Figure 5, the section of Maiyuqiao effectively exerting an influence during the positive flow period corresponded to the positive length of 26.5 km and the negative length of 7.2 km. Thus, the area influencing for the Maiyuqiao cross section was identified effectively.

**Figure 6:**
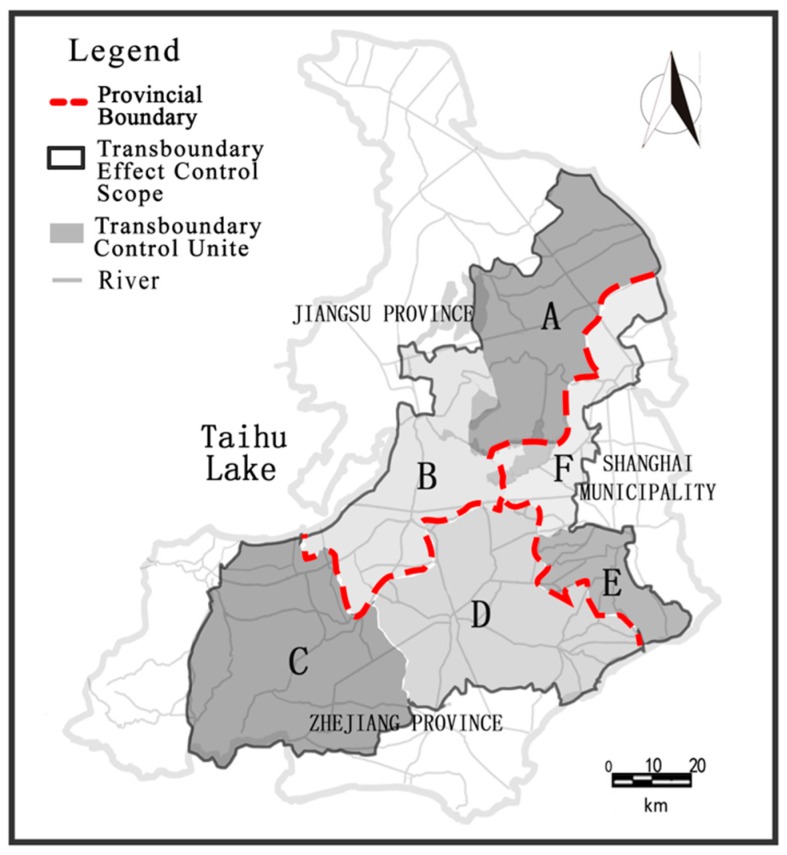
Trans-boundary effect control scope (TECS) and control units of the plain river network in Taihu Basin.

After superimposing the effectively influencing areas of the 31 important cross sections, the TECS boundary was delineated (Figure 6). The TECS will act as a joint management area for trans-boundary pollution control cooperation and collaboration. To maintain the effectiveness and ease of pollution control implementation, the scope was separated into six trans-boundary control units according to political administrative locations and targets of influence (Table 3).

### 4.2. Analysis of TACP

The Code of Practice for Computation on Allowable Permitted Assimilative Capacity of Water (GB/T 25173-2012) requires that the water environmental capacity is calculated using the discharge of the driest month under a 90% guarantee rate as the hydraulic condition. Based on hydrological data from 1955 to 2014, 1971 was identified as the year under the 90% guarantee. Therefore, the discharge of the driest month in 1971 was selected as the hydraulic boundary condition for the calculation. There is no exact requirement regarding water quality boundary conditions, thus water quality monitoring data from 2013 were selected.

In May 2010, the State Council of China approved “Taihu Lake Basin Water Environmental Function Zoning”, in which all important trans-boundary cross sections were required to achieve a standard of water quality equivalent to level III. According to the “Environmental Quality Standard for Surface Water” (GB3838-2002) [28] drafted by the Ministry of Environmental Protection of the People’s Republic of China in January 2002; the required water quality objectives are shown in Table 4. Once the boundary conditions and prescriptive targets have been identified, the environmental capacity of each trans-boundary control unit can be calculated.

Table 5 illustrates that the environmental capacity for COD_Cr_ in TECS is 160,806 t/year, with ammonia nitrogen, total nitrogen, and total phosphorus amounting to 16,098 t/year, 3493 t/year, and 39,768 t/year, respectively. The comparison with statistics on pollution sources in 2011 allows for the calculation of a pollution reduction rate (Table 5). The total amount of COD_Cr_ in the TECS must be reduced by 21.8% to guarantee that the water quality of the control objectives in the trans-boundary area reach the required standard, with ammonia nitrogen, total nitrogen, and total phosphorus needing reductions of 30.8%, 24.4%, and 7.4%, respectively. Compared with the defect rate of water quality in the corresponding districts collected in the twelve-five project, our pollution reduction rate indicates a similar spatial distribution in the six control units, which indicates that the result of the control calculation is reasonable.

## 5. Conclusions

As trans-boundary pollution and associated conflicts have frequently occurred in recent years, environmental departments worldwide are appealing for the adoption of an effective management methodology. In this paper, a new methodology regarding the consideration and control of total amount of pollutants in a trans-boundary river network region was put forward that highlights the delineation of the scope of the area to be managed, and the calculation of quantifiable pollution control targets.

Focusing on the complex trans-boundary pollution problem in the river network of Taihu Basin, an analysis of boundary water quality and domestic discharged pollutants was undertaken to survey current pollution in the study area. In order to propose a comprehensive and effective control management, the TECS was delineated based on effect analysis of various pollution sources, and the water environmental capacity of each of the proposed trans-boundary control units was calculated using the proposed trans-boundary water environmental capacity equation.

In order to achieve nationally stipulated levels of water quality, there needs to be a decrease of 21.8% in the amount of COD, 30.8% of NH_3_-N, 24.4% of TP, and 7.4% of TN from domestic pollutants discharged, in comparison to discharge volumes from 2011. The findings of this research will aid the administrative departments in the Taihu Basin in completing a comprehensive management strategy and designing a regional environmental policy. More importantly, the proposed method of trans-boundary pollution control, applicable for both the hilly river and plain river area, is generally helpful for identifying the priority control scope and the practical management of trans-boundary rivers. Through proper and targeted management for the trans-boundary environment, the pollution control process can be efficiently accelerated and pollution problem can be effectively addressed in these sensitive areas.

## Figures and Tables

**Table 1:** Administrative information of Study Area.

Province	City	Area (km^2^)	Number of Administrative Districts
Jiangsu	Suzhou	8488	5
Zhejiang	Huzhou (Partial)	5815	2
	Jiaxing	3915	6
Shanghai Municipality (Partial)		2649	5

**Table 2:** Comparison of the amount of pollutants discharged in the study area in 2011 and 2013.

Province	City	Amount of Pollution Discharged in River Network (t/year)							
		2011				2013			
		COD_Cr_	NH_3_-N	TP	TN	COD_Cr_	NH_3_-N	TP	TN
Jiangsu	Suzhou	158,068	22,453	2976	36,882	163,090	22,872	2598	35,005
Zhejiang	Jiaxing	107,389	12,235	2523	25,126	87,827	9968	1994	21,375
	Huzhou (partial)	88,949	8675	1752	18,341	83,321	8059	1696	17,192
Shanghai Municipality (partial)		52,429	6169	981	12,575	51,616	5959	942	11,750
SUM		406,835	49,532	9232	92,924	385,854	46,858	8130	85,322

COD_Cr_, chemical oxygen demand; NH_3_-N, ammonia nitrogen; TP, total phosphorus; TN, total nitrogen.

**Table 3:** Details of trans-boundary control units in the trans-boundary effect control scope (TECS).

Trans-Boundary Control Unit	Location	Main Target of Influence	Area (km^2^)
Unit A	Jiangsu Province	Jiangsu-Shanghai boundary	1465.5
Unit B	Jiangsu Province	Jiangsu-Zhejiang boundary	1461.2
Unit C	Zhejiang Province	Jiangsu-Zhejiang boundary	2085.9
Unit D	Zhejiang Province	Jiangsu-Zhejiang boundary Zhejiang-Shanghai boundary	2013.9
Unit E	Shanghai Municipality	Zhejiang-Shanghai boundary	516.2
Unit F	Shanghai Municipality	Jiangsu-Shanghai boundary	764.1

**Table 4:** Water quality objectives of trans-boundary cross sections.

Factors	Chemical Oxygen Demand (COD_Cr_)	Ammonia Nitrogen (NH_3_-N)	Total Phosphorus (TP)	Total Nitrogen (TN)
Concentration (mg/L)	20	1	0.2	1

**Table 5:** Total amount control of pollutant in the trans-boundary effective control area (TECS) of Taihu Basin.

Trans-Boundary Management Unit	Trans-Boundary Water Environmental Capacity (t/year)				Trans-Boundary Pollution Reduction Rate (%)			
	COD	NH_3_-N	TP	TN	COD	NH_3_-N	TP	TN
Unit A	32,475	2964	592	8010	27.60	26.80	15.90	2.40
Unit B	29,720	2769	554	7357	11.30	24.50	21.10	6.20
Unit C	39,669	4413	922	9888	23.80	33.40	19.50	4.70
Unit D	32,638	3262	875	7866	25.60	39.30	34.90	12.10
Unit E	10,606	1085	222	2680	14.50	23.80	17.10	10.40
Unit F	15,698	1605	328	3967	16.80	24.40	20.40	13.70
